# Peripheral circular RNAs hsa_circ_0075436 and hsa_circ_0005729 as diagnostic and prognostic biomarkers in acute ischemic stroke: expression profiles and mechanistic insights

**DOI:** 10.3389/fmolb.2025.1657284

**Published:** 2025-10-01

**Authors:** Wenqun Jiang, Weidong Chen, Shihao Lin, Pinpin Hou, Yichao Jin, Xiaohua Zhang, Hui Wu, Li Gao, Qin Hu

**Affiliations:** ^1^ Department of Laboratory Medicine, Ren Ji Hospital, School of Medicine, Shanghai Jiao Tong University, Shanghai, China; ^2^ Department of Anesthesiology, Punan Hospital in Pu Dong New District, Shanghai, China; ^3^ Department of Neurosurgery, Ren Ji Hospital, School of Medicine, Shanghai Jiao Tong University, Shanghai, China; ^4^ Shanghai Immune Therapy Institute, Ren Ji Hospital, School of Medicine, Shanghai Jiao Tong University, Shanghai, China; ^5^ Department of Neurology, Ren Ji Hospital, School of Medicine, Shanghai Jiao Tong University, Shanghai, China; ^6^ Key Laboratory of Anesthesiology, Shanghai Jiao Tong University, Ministry of Education, Shanghai, China

**Keywords:** acute ischemic stroke, circular RNA, peripheral blood mononuclear cell, whole transcriptome sequencing, biomarker

## Abstract

**Introduction:**

Circular RNAs (circRNAs) are a class of stable, conserved, and tissue-specific non-coding RNAs that have shown potential as diagnostic and prognostic biomarkers in acute ischemic stroke (AIS). However, their expression and functional roles in peripheral blood mononuclear cells (PBMCs) remain largely unexplored.

**Methods:**

We performed whole transcriptome RNA sequencing on PBMC samples from AIS patients (n = 5) and matched healthy controls (n = 5). The top 10 differentially expressed circRNAs were validated by qRT-PCR in a validation cohort (n = 60), and two consistently dysregulated circRNAs were further validated in a larger replication cohort (n = 288). Functional enrichment analysis and competing endogenous RNA (ceRNA) network construction were conducted to explore potential regulatory mechanisms.

**Results:**

Two circRNAs, hsa_circ_0075436 and hsa_circ_0005729, significantly decreased in PBMCs of AIS patients. The expressive levels negatively correlated with NIH Stroke Scale (NIHSS) scores at admission and discharge. Receiver operating characteristic (ROC) curve analysis demonstrated their strong diagnostic performance. Bioinformatics analyses and qRT-PCR further suggested that hsa_circ_0005729 may influence BBB disruption and peripheral immune suppression in AIS via hsa-miR-1301/COL1A1 axis.

**Conclusion:**

Hsa_circ_0075436 and hsa_circ_0005729 are promising PBMC-derived biomarkers for the diagnosis and prognosis of AIS.

## 1 Introduction

Acute ischemic stroke (AIS) results from a sudden interruption of cerebral blood flow. It is the second leading cause of death globally and a major contributor to adult disability, accounting for over 80% of all stroke cases (Bersano and Gatti, 2023). Accurate assessment of the extent and severity of brain injury is critical for guiding timely reperfusion therapies, such as intravenous thrombolysis and mechanical thrombectomy. Currently, evaluation of ischemic injury primarily relies on neuroimaging modalities, including magnetic resonance imaging (MRI) and computed tomography (CT). While essential, these techniques face notable limitations: restricted accessibility, variability in imaging protocols across institutions, and mismatch between imaging findings and clinical symptoms (Czap and Sheth, 2021; Ahmed et al., 2024). These challenges underscore the urgent need for more reliable, accessible, and objective diagnostic tools that can accurately assess cerebral injury and facilitate personalized treatment strategies.

In recent years, blood-based molecular biomarkers have gained attention for their potential in the diagnosis, prognosis, and prediction of complications of AIS (Cheng et al., 2024). Among them, circular RNAs (circRNAs), a newly identified class of non-coding RNAs, have shown particular promise. Unlike linear RNAs, circRNAs form covalently closed-loop structures without 5′caps or 3′poly-A tails, rendering them resistant to exonuclease degradation. This structural stability, together with their evolutionary conservation and tissue-specific expression patterns, makes circRNAs as attractive candidate biomarkers. Notably, blood-derived circRNAs such as circ-STAT3 and circTLK1 have been implicated in post-stroke inflammation and neuronal injury, suggesting both diagnostic and therapeutic relevance in AIS (Wu et al., 2019; Liu et al., 2021). Peripheral blood mononuclear cells (PBMCs), including monocytes, T cells, B cells, and natural killer (NK) cells, are central components of the immune system, characterized by a single round nucleus. Following stroke, disruption of the blood–brain barrier (BBB) activates the peripheral immune response, leading to PBMC infiltration into the ischemic brain and contributing to neuroinflammation and secondary brain injury (Moore et al., 2005). Despite their functional importance, the role of PBMC-derived circRNAs in AIS remains largely unexplored.

In this study, we aimed to identify and characterize circRNAs derived from PBMCs that may serve as biomarkers or therapeutic targets for AIS. Whole transcriptome sequencing (W.HTSeq) was performed on PBMCs from a discovery cohort of AIS patients (n = 5) and healthy controls (n = 5) to identify differentially expressed circRNAs (DECs). The top 10 DECs were selected for validation by quantitative real-time PCR (qRT-PCR) in a validation cohort (n = 60). Then, two circRNAs showing consistent dysregulation were chosen for further validation in an expanded, independent replication cohort (n = 288). Subsequently, a retrospective single-center case-control study involving both validation and replication cohorts (n = 348) was conducted to further assess the clinical relevance of the candidate circRNAs, with a focus on their diagnostic and prognostic value in AIS. To explore their potential biological functions, Gene Ontology (GO), Kyoto Encyclopedia of Genes and Genomes (KEGG) pathway, and protein–protein interaction (PPI) analyses were performed. Furthermore, competing endogenous RNA (ceRNA) networks were constructed to investigate the regulatory roles of the target circRNAs in AIS pathophysiology.

## 2 Materials and methods

### 2.1 Study population

In the discovery cohort, 5 patients with AIS and 5 age- and sex-matched healthy controls were recruited. PBMCs were isolated, and W. HTSeq was performed to identify DECs. For independent validation, an additional cohort comprising 174 AIS patients and 174 matched healthy controls was recruited. From this group, 30 individuals from each arm were randomly selected to form the validation cohort. The top 10 DECs identified in the discovery cohort were quantified using qRT-PCR to confirm expressive differences between AIS patients and healthy controls. To further assess the clinical relevance and diagnostic potential of the identified circRNA biomarkers, the remaining 144 AIS patients and 144 matched controls formed a replication cohort for extended analysis. Comprehensive demographic and clinical data were collected for all participants, including age, sex, fasting blood glucose, lipid profiles, and comorbidities such as hypertension, diabetes mellitus, and coronary artery disease. Stroke severity was evaluated using NIH Stroke Scale (NIHSS) scores at both admission and discharge.

Inclusion criteria were as follows: (a) first-ever AIS confirmed by head CT or MRI; (b) age between 18 and 85 years; and (c) presentation within 72 h of symptom onset, with stable vital signs upon evaluation.

Exclusion criteria included: (a) hemorrhagic stroke or other cerebrovascular diseases; (b) severe comorbid conditions, including but not limited to infections, hematological disorders, autoimmune diseases, and malignancies; and (c) patients who were unconscious, unable to cooperate, or unwilling to participate.

Healthy controls were recruited from individuals undergoing routine health examinations. They had no history of cerebrovascular events or major neurological disorders and were matched to AIS patients by age and sex.

### 2.2 Blood sample collection

All blood samples were collected immediately upon hospital admission, before the initiation of any therapeutic interventions. Whole blood was drawn into EDTA-K2 plasma collection tubes. To isolate PBMCs, 3.5 mL of whole blood was mixed with 6.5 mL of phosphate-buffered saline (PBS) to reach a total volume of 10 mL. The mixture was thoroughly homogenized. A 15 mL centrifuge tube was preloaded with 3.5 mL PBMC separation solution, corresponding to the original blood volume. The blood-PBS mixture was then gently layered onto the separation solution at a 45-degree angle. The tube was centrifuged at 1,200 *g* for 10 min at 4 °C. After centrifugation, the PBMC layer at the interface was carefully aspirated and transferred to a new 15 mL centrifuge tube. PBS was added to bring the volume up to 10 mL, followed by a second centrifugation at 600 *g* for 7 min.

### 2.3 Total RNA extraction

Total RNA was extracted from PBMCs using the RNA-Quick Purification Kit (Share-Bio, China) following the manufacturer’s instructions. An equal volume of absolute ethanol was added to the cell lysate and mixed thoroughly. The mixture was vigorously pipetted up and down 10 times, then transferred to a spin column. Next, 500 μL of Wash Buffer was added to the RNA column, followed by centrifugation at 12,000 × g for 1.5 min. The column was then placed into a clean, RNase-free 1.5 mL microcentrifuge tube and left to air-dry with the lid open for 2 min. To elute the RNA, 30 μL of Elution Buffer was added to the center of the column membrane, and the column was incubated at room temperature for 2 min, followed by centrifugation at 12,000 × g for 1 min to collect the eluted RNA. The RNA concentration was measured using a NanoDrop 2000 spectrophotometer (Thermo Scientific, United States of America) to ensure adequate quality for downstream analyses.

### 2.4 Whole transcriptome sequencing and data analysis

Total RNA was extracted and used to construct a ribosomal RNA (rRNA)-depleted library according to the manufacturer’s protocol (NEBNext Ultra Directional RNA Library Prep Kit). W. HTSeq was performed using a HiSeq™ Sequencer. Raw sequencing data were processed to remove adaptor sequences, reads containing more than 5% ambiguous bases (“N”), and low-quality reads in which over 20% of bases had quality scores below 20. The high-quality filtered reads were then aligned to the human reference genome (GRCh38, NCBI) using HISAT2 (Kim et al., 2015). Gene counts for messenger RNA (mRNA) and long non-coding RNA (lncRNA) were calculated using the HTSeq framework (Anders et al., 2015).

### 2.5 CircRNA prediction and data analysis

CircRNAs were predicted from the sequencing data using the ACFS circRNA pre-diction pipeline (You et al., 2015). Unmapped reads were further processed with BWA-MEM (bwa mem -t 1 -k 16 -T 20) to identify potential circRNAs. Head-to-tail (back-splicing) junctions were detected, and splicing strength scores were calculated using MaxEntScan, with a filtering threshold of ≥10. To quantify circRNAs, unmapped reads were realigned to the predicted circRNA candidates. Reads mapping to the back-splice junctions, with a minimum overhang of 6 nucleotides, were counted for each circRNA.

### 2.6 Differentially expressed gene analysis and circRNA-related ceRNA network construction

Differential expression analysis of genes—including mRNAs, lncRNAs, and circRNAs—was performed using EBSeq (v1.16.0). The following criteria were used to identify differentially DECs: fold-change ≥1.5 and false discovery rate (FDR) < 0.05, with multiple testing correction implemented via the Benjamini–Hochberg procedure. MiRNA targets were predicted using the Miranda and RNAhybrid algorithms, focusing on the 3′untranslated regions (UTRs) of differentially expressed mRNAs and the full-length sequences of DECs. Based on shared miRNA interactions, a circRNA-related ceRNA network was constructed by linking circRNAs to mRNAs regulated by the same miRNAs, thus revealing potential ceRNA regulatory relationships.

### 2.7 Functional analysis

Functional annotation was conducted using the GO database (downloaded from NCBI, UniProt, and AmiGO), and pathway analysis was performed using the KEGG database. Statistical significance was assessed using Fisher’s exact test, and the FDR was calculated using the Benjamini–Hochberg method.

### 2.8 Validation of DECs and target genes with qRT-PCR

The results of W. HTSeq and circRNA regulatory target genes predictions were validated by qRT-PCR. Complementary DNA (cDNA) was synthesized from total RNA extracted from PBMCs using the Evo M-MLV RT Kit (Accurate Biotechnology, Changsha, China) according to the manufacturer’s instruction. qRT-PCR was performed on the QuantStudio™ 5 Real-Time PCR System (Thermo Fisher Scientific, MA, United States of America) using the SYBR Green Premix Pro Taq HS qPCR Kit (Accurate Biotechnology, Changsha, China). For circRNA analysis, each qRT-PCR reaction was conducted in a total volume of 10 μL, comprising 5 μL of 2× SYBR Green Pro Taq HS Premix, 0.2 μL of forward primer (10 μM), 0.2 μL of reverse primer (10 μM), 2 μL of cDNA template, and 2.6 μL of RNase-free water. The optimized cycling conditions were initial denaturation at 95 °C for 30 s, followed by 40 cycles of 95 °C for 5 s and 60 °C for 30 s. Melting curve analysis revealed a single peak, confirming the specificity of the amplification. Relative gene expression levels were calculated using Glyceraldehyde-3-phosphate dehydrogenase (GAPDH) as the internal control. All qRT-PCR primers were designed and synthesized by Accurate Biotechnology (Changsha, China).

### 2.9 ROC curve analysis

Based on the qRT-PCR results, the relative expression levels of circRNAs—normalized using the 2^−ΔΔCt^ method—were used as predictor variables, with clinical diagnosis (AIS = 1, healthy control = 0) serving as the response variable. ROC curve analysis was performed to assess the diagnostic performance of the circRNAs. In the ROC curve, the x-axis represents the false positive rate (FPR), calculated as FPR = FP/(FP + TN), where FP is the number of false positives and TN is the number of true negatives. The y-axis represents the true positive rate (TPR or recall), calculated as TPR = TP/(TP + FN), where TP is the number of true positives and FN is the number of false negatives. The discriminative power of each circRNA was quantified by the AUC. An AUC of 0.5 indicates no diagnostic ability (equivalent to random chance), while an AUC of 1.0 indicates perfect discrimination. The qRT-PCR data were analyzed using the ROC curve module in GraphPad Prism, which provided the AUC values along with sensitivity, specificity, and the corresponding 95% confidence intervals.

### 2.10 Cell culture

HEK293T cells used in this study were purchased from Wuhan Servicebio Technology Co., Ltd. and authenticated by the supplier. Cells were maintained in high-glucose Dulbecco’s Modified Eagle’s Medium (DMEM; Share-Bio, China) supplemented with 10% fetal bovine serum (FBS; Servicebio, China). Cultures were incubated at 37 °C in a humidified atmosphere containing 5% CO_2_.

### 2.11 Cell transfection

HEK293T cells were seeded into 6-well plates and cultured until reaching approximately 70% confluence. Recombinant plasmids and miRNA mimics were co-transfected using Hieff Trans liposomal transfection reagent (YEASEN, China) according to the manufacturer’s instructions. After 48 h of transfection, the cells were harvested for subsequent analyses.

### 2.12 Dual-luciferase reporter assay

Predicted wild-type (WT) and mutant (MUT) binding sites of hsa-miR-1301-3p within the hsa_circ_0005729 sequence or the COL1A1 3′UTR were synthesized and cloned into the pmirGLO dual-luciferase miRNA target expression vector, generating hsa_circ_0005729-WT/MUT and COL1A1-WT-3′UTR/MUT-3′UTR constructs (Sangon Biotech, China). The recombinant plasmids were co-transfected with either hsa-miR-1301-3p mimics or negative control (NC) mimics (Sangon Biotech, China; Supplementary Table S1) into HEK293T cells. After 48 h, relative luciferase activity was measured using a Dual-Luciferase Reporter Assay Kit (Beyotime, China) following the manufacturer’s protocol.

### 2.13 Statistical analysis

Statistical analyses were conducted using GraphPad Prism version 9.0 or SPSS 22.0. DECs were defined by an absolute FC ≥ 1.5 and a *P*-value <0.05. Normally distributed continuous variables are presented as mean ± standard deviation (x̄ ± s), while non-normally distributed data are expressed as medians with IQRs (M [Q1, Q3]). The Mann–Whitney U test was used to compare non-normally distributed variables. Categorical variables were analyzed using the χ^2^ test and are reported as counts and percentages. For correlation analysis, Pearson’s correlation coefficient was used for continuous variables, and Spearman’s rank correlation was applied to ordinal data. Univariate and multivariate logistic regression analyses were performed to identify independent predictors of AIS. Variables with a *P*-value <0.10 in the univariate analysis were included in the multivariate model. Adjusted odds ratios (ORs) and corresponding 95% confidence intervals (CIs) were calculated to assess the strength of associations. Relative quantification of DECs was performed using the 2^−ΔΔCt^ method. A *P*-value <0.05 was considered statistically significant.

## 3 Results

### 3.1 Expression profiles of circRNAs in PBMCs after AIS

**FIGURE 1:**
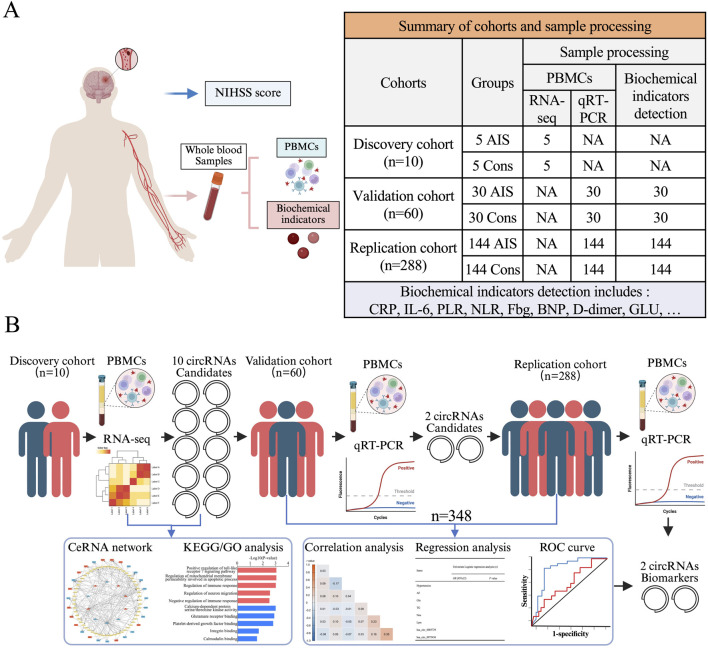
The samples acquisition process and simplified flowchart of circRNA biomarker discovery. **(A)** Whole blood samples were acquired from all recruited patients followed by PBMCs isolation and biochemical indicators detection, while NIHSS scores were requested only in AIS patients. **(B)** In this report, a discovery cohort was prospectively recruited to identify specific circRNA molecules in PBMCs of AIS patients via W. HTSeq. A validation cohort and a replication cohort were retrospectively analyzed for verification and correlation with AIS-related indicators.

The sample acquisition process and a simplified workflow for circRNA biomarker identification are illustrated in Figures 1A,B. The discovery cohort included 5 AIS patients and 5 age- and sex-matched healthy controls. Their demographic and clinical characteristics are summarized (Supplementary Table S2).

**FIGURE 2:**
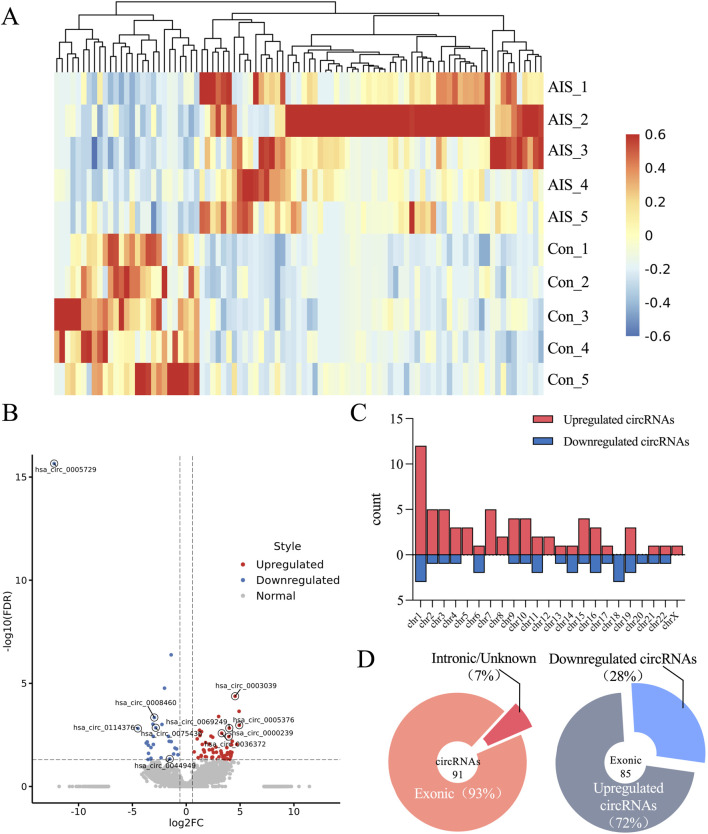
Differentially expressed CircRNA profiles in AIS patients and control subjects. **(A)** Heat map showing circRNAs differentially expressed between AIS and control groups. **(B)** Volcano plot illustrating circRNA expression profiles in AIS and control groups. Red dots represent significantly upregulated circRNAs (FC ≥ 1.5 and *P* < 0.05, n = 5 per group, two-sample t-test). Blue dots represent significantly downregulated circRNAs (FC ≤ −1.5 and *P* < 0.05, n = 5 per group, two-sample t-test). **(C)** Bar chart displaying the chromosomal distribution of DECs. **(D)** Pie chart illustrating the genomic origin of DECs in AIS patients, categorized as exonic, intronic, or unknown. “Exonic” indicates that both ends of the back-splice junction are located within exons; “Intronic” indicates that both ends are located within introns, or one end lies outside the exon region; “Unknown” refers to cases where the back-splice site does not overlap with any annotated transcript.

Based on W. HTSeq results and applying the criteria of fold change (FC) ≥ 1.5 and *P* < 0.05, a total of 91 DECs were identified, including 64 upregulated and 27 downregulated circRNAs (Figures 2A,B). The majority of these circRNAs were derived from exonic regions and distributed across multiple chromosomes (Figures 2C,D). The top 10 DECs, ranked by log_2_ FC, are presented in Table 1 along with their corresponding *P*-values.

**TABLE 1:** The top 10 of differentially DECs in patients with AIS ranked by log2 (FC).

CircRNA ID	GeneName	Log2 (FC)	*P*	Regulate
hsa_circ_0005376	HOOK3	4.923487	0.00107378	up
hsa_circ_0003039	ATF6	4.53764	0.00004219	up
hsa_circ_0069249	QDPR	3.998159	0.00147723	up
hsa_circ_0000239	RUFY2	3.954696	0.00380933	up
hsa_circ_0036372	UBE2Q2	3.295542	0.00261897	up
hsa_circ_0005729	RNF138	−12.2647	2.2204E-16	down
hsa_circ_0114376	PKN2	−4.48923	0.00154408	down
hsa_circ_0044949	USP3	−3.08926	0.00095338	down
hsa_circ_0008460	WHSC1	−2.96388	0.00045393	down
hsa_circ_0075436	EXOC2	−2.83092	0.00142166	down

FC, fold change.

**FIGURE 3:**
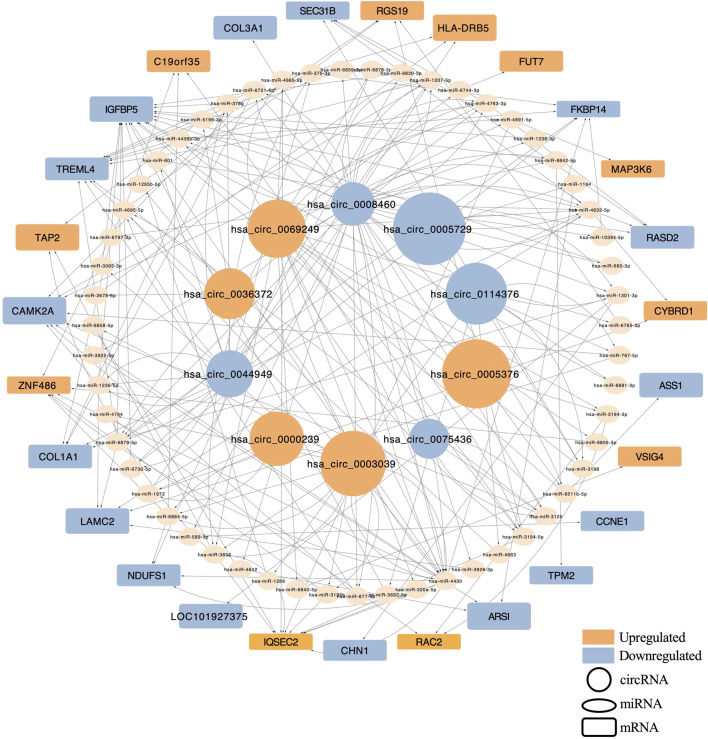
The Network of circRNA-miRNA-mRNA of the top 10 differentially quantified circRNAs. Circular nodes represented circRNAs, elliptical nodes denoted miRNAs, and rounded rectangles indicated mRNAs. Red indicated upregulation, while blue indicated downregulation. The size of circular nodes and rounded rectangles corresponded to the expression variation (log_2_ FC) of circRNAs and their target mRNAs.

To investigate the regulatory potential of these circRNAs, a ceRNA (circRNA–microRNA (miRNA)–mRNA) network was constructed for the top 10 DECs. Predicted miRNA interactions were obtained using Miranda and RNAhybrid algorithms, with network edges defined by shared miRNA binding sites. The resulting network included 55 miRNAs and 27 target mRNAs, comprising 109 interaction pairs (Figure 3). Among these, hsa_circ_0008460 showed the highest degree of connectivity, interacting with 20 miRNAs and regulating 12 mRNAs through 42 interaction pairs. KEGG and GO pathway enrichment analyses indicated that the host genes of dysregulated circRNAs are involved in multiple AIS-related biological processes, including neural development, immune response, protein degradation, cell motility, and cell cycle regulation (Supplementary Figures S1, S2). These findings provide mechanistic insight into the potential roles of circRNAs in AIS pathophysiology.

### 3.2 Validation of DECs in validation cohort by qRT-PCR

**FIGURE 4:**
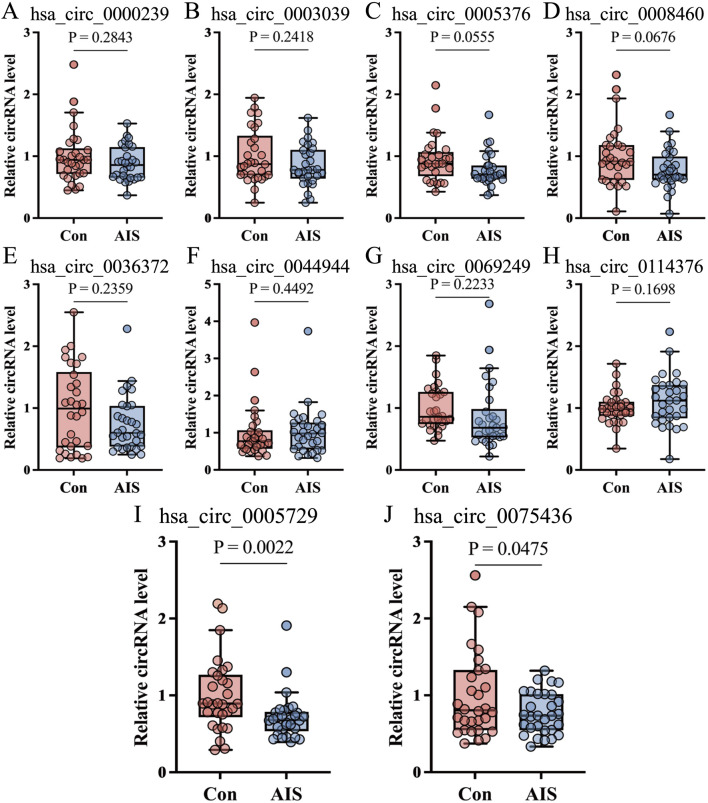
qRT-PCR validation of ten candidate circRNAs in validation cohort. **(A–J)** qRT-PCR confirmed the sequencing results for selected circRNAs—five upregulated and five downregulated—in AIS patients compared with control subjects (n = 30 per group), as shown in the boxplots. Boxplots showed median (central line), interquartile range (IQR, box boundaries), with whiskers extending to 1.5 × IQR. Points beyond whiskers were shown as outliers. Statistical significance was determined by Mann-Whitney U test. AIS, acute ischemic stroke; circRNA, circular RNA; qRT-PCR, quantitative real-time polymerase chain reaction.

A total of 174 AIS patients and 174 age- and sex-matched healthy controls were recruited. Baseline clinical data of the whole samples are shown in Table 2. From this cohort, 30 subjects from each group were randomly selected to form the validation cohort. qRT-PCR was performed to validate the expression of the top 10 DECs, including five upregulated circRNAs (hsa_circ_0005376, hsa_circ_0003039, hsa_circ_0069249, hsa_circ_0000239, and hsa_circ_0036372) and five downregulated circRNAs (hsa_circ_0005729, hsa_circ_0114376, hsa_circ_0044949, hsa_circ_0008460, and hsa_circ_0075436). Primer sequences are provided in Supplementary Table S3. Among these, hsa_circ_0005729 and hsa_circ_0075436 were significantly downregulated in AIS patients compared to controls (Figures 4A–J).

**TABLE 2:** Baseline clinical characteristics of the validation and replication samples.

Characteristics	AIS (*n* = 174)	Control (*n* = 174)	*t/χ^2^/Z* value	*P* Value
Age (mean ± SD), year	65.24 ± 11.44	65.51 ± 8.60	−0.249	0.804
Male, n (%)	120 (69.0%)	128 (73.6%)	0.898	0.343
Hypertension, n (%)	122 (70.11%)	55 (31.6%)	51.613	0.000
Coronary heart disease, n (%)	8 (4.6%)	7 (4.0%)	0.070	0.792
AF, n (%)	18 (10.3%)	1 (0.6%)	16.089	0.000
GLU (mmol/L)	8.00 ± 8.42	5.59 ± 1.14	3.740	0.000
TC (mmol/L)	4.72 ± 1.16	4.87 ± 0.87	−1.400	0.162
TG (mmol/L)	1.99 ± 2.63	1.52 ± 0.84	2.261	0.024
LDL (mmol/L)	3.03 ± 0.87	3.12 ± 0.68	−1.027	0.305
Neu (10^9^/L)	5.65 ± 2.53	3.51 ± 1.12	10.127	0.000
Lym (10^9^/L)	1.68 ± 0.81	2.02 ± 0.62	−4.360	0.000
Mono (10^9^/L)	0.48 ± 0.20	0.46 ± 0.14	1.300	0.195
CREA (umol/L)	75.03 ± 24.73	76.87 ± 14.46	−0.847	0.398
CysC (mg/L)	1.02 ± 0.35	0.98 ± 0.16	1.427	0.155
PLT (10^9^/L)	203.39 ± 61.94	213.41 ± 52.84	−1.625	0.105
NLR	4.05 ± 2.80	1.90 ± 0.88	9.68	0.000
PLR	141.62 ± 68.41	114.29 ± 45.23	4.40	0.000
NIHSS score	7.20 ± 5.38	—	—	—
CRP (mg/L)	8.82 ± 21.62	—	—	—
IL-6 (pg/mL)	26.57 ± 85.50	—	—	—
BNP (pg/mL)	115.21 ± 253.09	—	—	—
D-Dimer (ug/mL)	0.50 ± 1.18	—	—	—
Fbg (g/L)	3.13 ± 0.86	—	—	—

Values are expressed as mean ± standard deviation or n (%). AF, atrial fibrillation; GLU, glucose; TC, total cholesterol; TG, triglycerides; LDL, low density lipoprotein; Neu, Neutrophil; Lym, lymphocyte; Mono, Monocytes; CREA, creatinine; CysC, Cystatin C; PLT, platelets; NLR, Neutrophil-to-lymphocyte ratio; PLR, Platelet-to-lymphocyte ratio; CRP, C-reactive protein; IL-6, Interleukin-6; BNP, B-type natriuretic peptide; Fbg, Fibrinogen.

### 3.3 Downregulation of hsa_circ_0005729 and hsa_circ_0075436: diagnostic and prognostic potential

**FIGURE 5:**
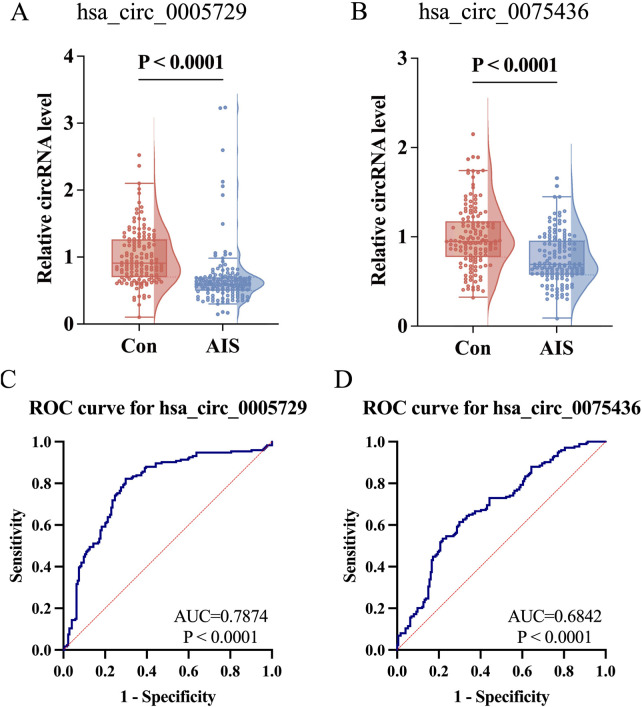
Further validation of hsa_circ_0075436 and hsa_circ_0005729 in replication cohort (n = 288) and ROC curve analysis in the combined cohort (n = 348). **(A,B)** Expression levels of hsa_circ_0005729 and hsa_circ_0075436 in AIS patients and matched controls (n = 144 per group). Boxplots displayed the median (central line), interquartile range (IQR; box boundaries), with whiskers extending to 1.5 × IQR. Points beyond whiskers were shown as outliers. Violin plots showed the distribution of the data. Statistical significance was determined by the Mann-Whitney U test. **(C,D)** ROC curve analysis of the two validated circRNAs in the combined cohort (n = 348). The red diagonal line in each graph represents the reference line for no diagnostic discrimination (AUC = 0.500). AIS, acute ischemic stroke; circRNA, circular RNA; qRT-PCR, quantitative real-time polymerase chain reaction; ROC, Receiver operating characteristic.

To further validate the expression changes of hsa_circ_0005729 and hsa_circ_0075436 in AIS patients, qRT-PCR was performed in an independent replication cohort consisting of remaining 144 AIS patients and 144 matched controls. Representative amplification and melt curve plots from qRT-PCR analysis are provided (Supplementary Figures S3, S4). The qRT-PCR results confirmed that both circRNAs were significantly downregulated in AIS patients, reinforcing their association with AIS (Figures 5A,B). To evaluate their diagnostic potential, ROC curve analysis was performed. Hsa_circ_0005729 demonstrated superior diagnostic performance with an area under the curve (AUC) of 0.7874 (95% CI = 0.7379 to 0.8368, *P* < 0.0001), achieving sensitivity of 82.18% and specificity of 70.11% at a cut off value of <0.8399 (Figure 5C). Although hsa_circ_0075436 yielded a lower AUC of 0.6842 (95%CI = 0.6284 to 0.7399, *P* < 0.0001), it still demonstrated clinically relevant discriminatory power, with 53.45% sensitivity and 78.16% specificity at a cut off value of <0.7647 (Figure 5D).

**FIGURE 6:**
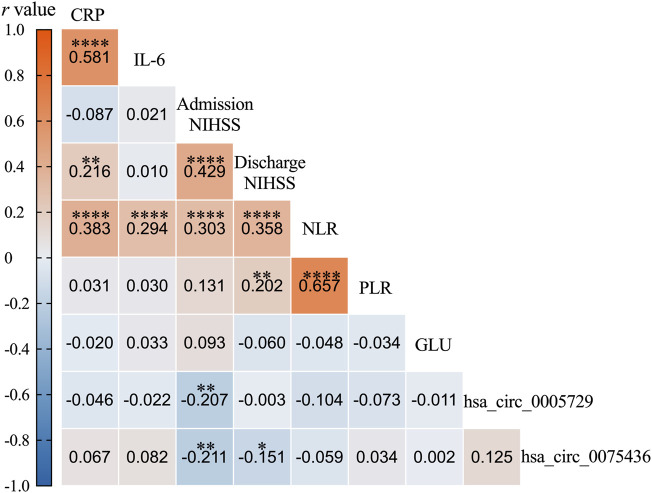
The heatmap illustrated the correlation between circRNA expression and clinical parameters. Red indicated a strong positive correlation (closer to +1), while blue indicated a strong negative correlation (closer to −1). White denoted a correlation near zero. *P*-values were indicated as follows: *P < 0.05; **P < 0.01; ***P < 0.001; ****P < 0.0001 (two-tailed test). NLR: Neutrophil-to-lymphocyte ratio; PLR: Platelet-to-lymphocyte ratio; CRP: C-reactive protein; IL-6: Interleukin-6; GLU: Glucose.

Correlation analyses revealed that both circRNAs were negatively associated with NIHSS scores at admission (hsa_circ_0005729: r = −0.207, 95%CI = −0.3452 to −0.0592, *P* = 0.006; hsa_circ_0075436: r = −0.211, 95%CI = −0.3489 to −0.063, *P* = 0.005). Hsa_circ_0075436 was also negatively correlated with NIHSS scores at discharge (r = −0.151, 95%CI = −0.2933 to −0.002 *P* = 0.047). The neutrophil-to-lymphocyte ratio (NLR) was positively correlated with both admission NIHSS scores and discharge NIHSS scores (r = 0.303, 95%CI = 0.1607 to 0.4324; r = 0.358, 95%CI = 0.2212 to 0.4813, both *P* < 0.0001), and platelet-lymphocyte ratio (PLR) levels was also positively associated with discharge NIHSS scores (r = 0.202, 95%CI = 0.0552 to 0.3408, *P* = 0.007). Given that elevated blood glucose levels were consistently observed in AIS patients compared to controls across three cohorts, this metabolic parameter was included as a covariate in the correlation analysis to evaluate its potential influence on hsa_circ_0005729 and hsa_circ_0075436 expression. The results showed no significant association between blood glucose levels and either circRNA (hsa_circ_0005729: r = −0.011, 95%CI = −0.1595 to 0.1380, *P* = 0.8858; hsa_circ_0075436: r = 0.002, 95% CI = −0.1473 to 0.1503, *P* = 0.9841) (Figure 6).

### 3.4 Risk factors for AIS

Univariate and multivariate logistic regression analyses were performed on the combined cohort (n = 348) to identify independent risk factors associated with AIS (Table 3). Univariate logistic regression analysis identified several variables significantly associated with AIS: hypertension (OR = 5.076, 95%CI = 3.219 to 8.005, *P* < 0.001), atrial fibrillation (OR = 19.962, 95%CI = 2.634 to 151.271, *P* = 0.004), elevated blood glucose (OR = 1.711, 95%CI = 1.451 to 2.016, *P* < 0.001), elevated triglycerides (OR = 1.251, 95%CI = 1.014 to 1.544, *P* = 0.037), increased neutrophil counts (OR = 2.169, 95%CI = 1.785 to 2.636, *P* < 0.001), decreased lymphocyte counts (OR = 0.497, 95%CI = 0.360 to 0.688, *P* < 0.001), and reduced expression of hsa_circ_0005729 (OR = 0.130, 95%CI = 0.067 to 0.253, *P* < 0.001) and hsa_circ_0075436 (OR = 0.131, 95%CI = 0.066 to 0.262, *P* < 0.001). Multivariate logistic regression further confirmed that hypertension (OR = 5.663, 95%CI = 2.899–11.063), atrial fibrillation (OR = 9.725, 95%CI = 1.084–87.251), elevated blood glucose (OR = 1.441, 95%CI = 1.181–1.758), increased neutrophil counts (OR = 2.177, 95%CI = 1.689–2.805), decreased lymphocyte counts (OR = 0.443, 95%CI = 0.281–0.696), and reduced levels of hsa_circ_0005729 (OR = 0.224, 95%CI = 0.109–0.459) and hsa_circ_0075436 (OR = 0.143, 95%CI = 0.052–0.397) were independent risk factors for AIS (all *P* < 0.001, except atrial fibrillation, *P* = 0.042). Triglyceride levels were not significant in the multivariate model (*P* = 0.093).

**TABLE 3:** Univariate and Multivariate Logistic regression analysis of AIS.

Items	Univariate logistic regression analysis (*n* = 348)		Multivariate logistic regression analysis (*n* = 348)	
	OR (95%*CI*)	*P* Value	Adjusted *OR* (95%*CI*)	*P* Value
Hypertension	5.076 (3.219, 8.005)	<0.001	5.663 (2.899, 11.063)	<0.001
AF	19.962 (2.634, 151.271)	0.004	9.725 (1.084, 87.251)	0.042
Glu	1.711 (1.451, 2.016)	<0.001	1.441 (1.181, 1.758)	<0.001
TG	1.251 (1.014, 1.544)	0.037	1.203 (0.969, 1.494)	0.093
Neu	2.169 (1.785, 2.636)	<0.001	2.177 (1.689, 2.805)	<0.001
Lym	0.497 (0.360, 0.688)	<0.001	0.443 (0.281, 0.696)	<0.001
hsa_circ_0005729	0.130 (0.067, 0.253)	<0.001	0.224 (0.109, 0.459)	<0.001
hsa_circ_0075436	0.131 (0.066, 0.262)	<0.001	0. 143 (0.052, 0.397)	<0.001

CI, confidence interval; OR, odds ratio; AF, atrial fibrillation; GLU, glucose; TG, triglycerides; Neu, Neutrophil; Lym, lymphocyte.

### 3.5 GO and KEGG pathway analysis of circRNAs target genes

**FIGURE 7:**
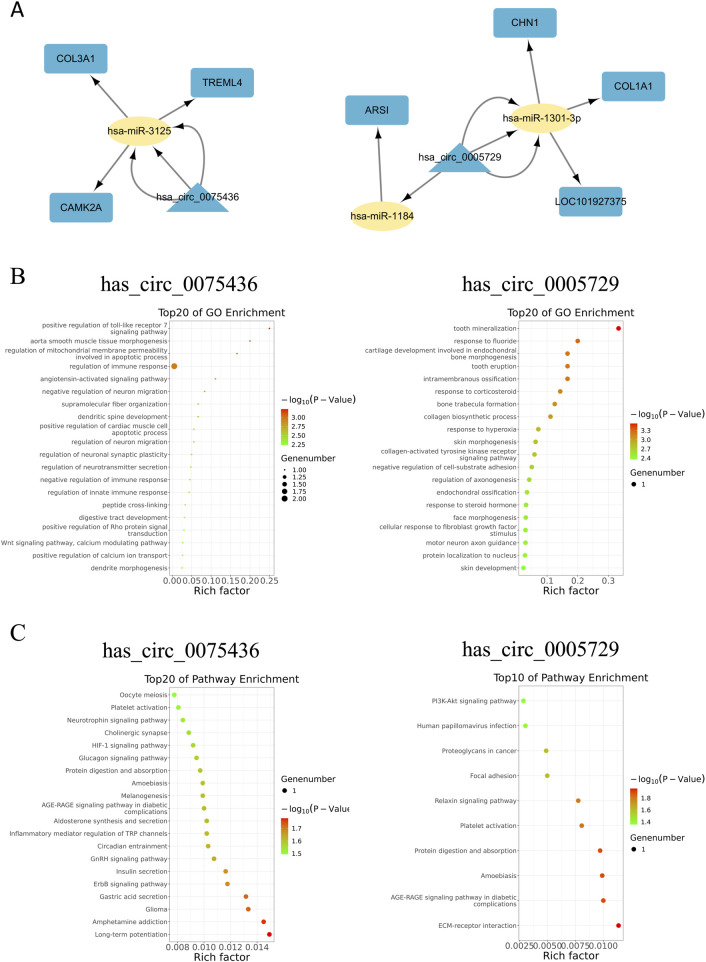
Functional prediction of hsa_circ_0075436 and hsa_circ_0005729 based on circRNA-miRNA-mRNA network. **(A)** circRNA–miRNA–mRNA interaction network for hsa_circ_0075436 and hsa_circ_0005729. Triangles represent circRNAs, ellipses represent miRNAs, and rounded rectangles represent mRNAs. **(B)** GO-analysis of putative target genes of hsa_circ_0075436 and hsa_circ_0005729. **(C)** KEGG pathway analysis of putative target genes of hsa_circ_0075436 and hsa_circ_0005729.

To elucidate the functional roles of hsa_circ_0075436 and hsa_circ_0005729 in AIS, circRNA–miRNA–mRNA networks were constructed using Miranda and RNAhybrid algorithms (Figure 7A). Seven regulatory axes involving three key mRNAs were identified: hsa_circ_0075436–hsa-miR-3125–CAMK2A, hsa_circ_0075436–hsa-miR-3125–COL3A1, hsa_circ_0075436–hsa-miR-3125–TREML4, hsa_circ_0005729–hsa-miR-1301–CHN1, hsa_circ_0005729–hsa-miR-1301–COL1A1, hsa_circ_0005729–hsa-miR-1301–LOC101927375, and hsa_circ_0005729–hsa-miR-1184–ARSI. These regulatory interactions suggest that both circRNAs may influence AIS pathophysiology through modulation of key gene expression networks. The mRNAs significantly regulated by hsa_circ_0075436 and hsa_circ_0005729 are listed in Supplementary Table S4, together with their log_2_FC values and false discovery rates (FDRs).

Functional enrichment analyses suggested that hsa_circ_0075436 and hsa_circ_0005729 may modulate stroke-relevant pathways through distinct mechanisms. For hsa_circ_0075436, GO analysis revealed significant enrichment in toll-like receptor signaling, regulation of mitochondrial membrane permeability, immune response, dendritic spine development, and neuron migration (Figure 7B). KEGG pathway analysis further indicated involvement in long-term potentiation, ErbB signaling, and TRP channel regulation by inflammatory mediators (Figure 7C), highlighting potential roles in immune signaling, synaptic plasticity, inflammation, and neuronal survival. In contrast, hsa_circ_0005729 was enriched in GO terms related to collagen biosynthesis, response to hyperoxia, tyrosine kinase receptor signaling, axonogenesis, and motor neuron guidance (Figure 7B). KEGG analysis identified enrichment in ECM–receptor interaction, platelet activation, and the PI3K–Akt signaling pathway (Figure 7C), suggesting potential involvement in extracellular matrix remodeling, vascular regulation, and cell survival pathways.

### 3.6 Validation of hsa_circ_0075436 and hsa_circ_0005729 target genes

**FIGURE 8:**
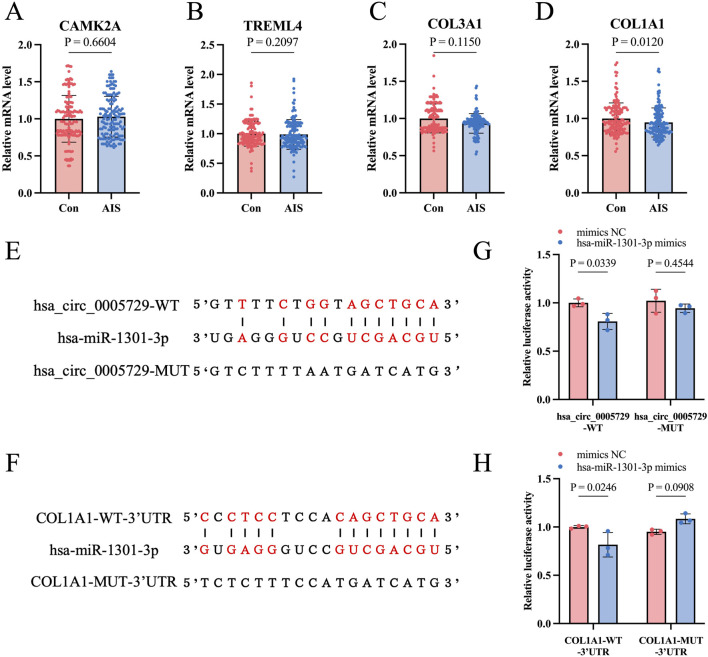
Validation of hsa_circ_0005729 and hsa_circ_0075436 target genes and verification of the hsa_circ_0005729/miR-1301-3p/COL1A1 ceRNA axis. **(A–D)**. The mRNA levels of hsa_circ_0005729 and hsa_circ_0075436 target genes (CAMK2A, TREML4, COL1A1, COL3A1). Statistical significance was determined by the Mann-Whitney U test. **(E)** Predicted binding sites between hsa_circ_0005729 and hsa-miR-1301-3p. **(F)** Predicted binding sites between COL1A1 3′UTR and hsa-miR-1301-3p. **(G)** Luciferase activity of hsa_circ_0005729-WT and hsa_circ_0005729-MUT reporters in HEK293T cells co-transfected with hsa-miR-1301-3p mimics or negative control (NC) mimics. **(H)** Luciferase activity of COL1A1-WT-3′UTR and COL1A1-MUT-3′UTR reporters in HEK293T cells co-transfected with hsa-miR-1301-3p mimics or NC mimics.

We validated the mRNA levels of CAMK2A, TREML4, COL3A1, and COL1A1—predicted target genes of hsa_circ_0075436 and hsa_circ_0005729 implicated in inflammatory responses and matrix remodeling—using qRT-PCR in the replication cohort. Primer sequences are provided in Supplementary Table S5. The results showed that COL1A1 mRNA levels were significantly reduced in AIS patients compared with controls, consistent with the transcriptomic data (Figures 8A–D). Bioinformatic analysis using RNAhybrid predicted the presence of hsa-miR-1301-3p binding sites within the hsa_circ_0005729 sequence (Figure 8E). In addition, hsa-miR-1301-3p was also predicted to directly target COL1A1, consistent with our earlier analysis (Figure 8F). Dual-luciferase reporter assays further validated these predictions: transfection with hsa-miR-1301-3p mimics in HEK293T cells significantly reduced the luciferase activity of the hsa_circ_0005729-WT and COL1A1-WT-3′UTR reporters, whereas no significant effect was observed for the hsa_circ_0005729-MUT and COL1A1-MUT-3′UTR constructs (Figures 8G,H).

## 4 Discussion

In recent years, circRNAs have emerged as promising molecular biomarkers for the diagnosis and prognosis of a wide range of diseases, owing to their high stability, evolutionary conservation, and tissue-specific expression profiles. Advances in high-throughput sequencing have further accelerated circRNA discovery and functional characterization (Wen et al., 2021). In this single-center cohort study, we investigated the potential of circRNAs derived from PBMCs as diagnostic and prognostic biomarkers for AIS. Among the identified candidates, hsa_circ_0075436 and hsa_circ_0005729 were significantly downregulated in AIS patients. Their expression levels were inversely correlated with NIHSS scores at both admission and discharge, and ROC curve analysis demonstrated their potential diagnostic value. Bioinformatics analysis suggested that these circRNAs may act as miRNA sponges, regulating key genes through the hsa-miR-3125/CAMK2A, hsa-miR-3125/TREML4, hsa-miR-3125/COL3A1, and hsa-miR-1301/COL1A1 axes, thereby highlighting their potential as therapeutic targets.

After ischemic stroke, the brain tissue subjected to hypoxia and ischemia undergoes necrosis, releasing damage-associated molecular patterns (DAMPs) such as HMGB1, heat shock proteins, and ATP (Qin et al., 2022; Ding et al., 2023; Ge, 2024; Xu et al., 2024). These DAMPs activate Toll-like receptors and the NLRP3 inflammasome, initiating a cascade of inflammatory responses. The impaired BBB allows inflammatory factors to enter the peripheral circulation and activate PBMCs, leading to widespread changes in gene expression, including circRNA expression (Huang et al., 2021; Beylerli et al., 2024). Furthermore, circRNAs released from damaged brain tissue may be taken up by PBMCs (Jiang et al., 2024). Owing to their high stability, resistance to exonuclease degradation, and widespread presence in peripheral blood, circRNAs have emerged as promising biomarker candidates. Recent studies have shown that circRNAs can be reliably detected in PBMCs, with several identified as potential diagnostic markers for autoimmune diseases such as myasthenia gravis (Wang et al., 2024). Moreover, accumulating evidence indicates that PBMC-derived circRNAs display significant expression changes following AIS (Li et al., 2025), many of which are associated with inflammation, immune responses, neuronal injury, and tissue repair (Liu et al., 2022). Given the accessibility of PBMCs through routine venipuncture and the inherent stability of circRNAs, PBMC-derived circRNAs represent a minimally invasive and clinically feasible approach for monitoring AIS-related molecular alterations and assessing disease severity. By contrast, although serum and plasma samples are more readily accessible than PBMCs, our research experience suggests that high-throughput differential screening in these fluids often yields limited identification of suitable circRNA candidates for subsequent validation and modeling in acute ischemic stroke.

CircRNAs regulate gene expression via various mechanisms, most notably by acting as miRNA sponge to prevent miRNAs from binding their target mRNAs. They can also act as scaffolds for RNA-binding proteins, influencing protein function and stability. Among the DECs, hsa_circ_0075436 and hsa_circ_0005729 stood out for their predicted interactions with hsa-miR-3125, hsa-miR-1301-3p, and hsa-miR-1184. These miRNAs are involved in a variety of pathophysiological processes. For instance, hsa-miR-3125 has been found to be downregulated in glioblastoma patients with poor survival, suggesting a protective role in cancer biology (Hermansen et al., 2017). However, it is upregulated in immune thrombocytopenia and induce platelet apoptosis and adhesion (Deng et al., 2017). Hsa-miR-1301-3p plays a tumor-suppressive role in several cancers by regulating cell proliferation, apoptosis, and migration, targeting genes such as SRR and modulating key signaling pathways like MAPK and PI3K-Akt (Zheng et al., 2021; Cui et al., 2022). Similarly, hsa-miR-1184 is downregulated in osteosarcoma and colorectal cancer, where it aggravates the progression of tumor by enhancing lipid metabolism and activating the Hippo/YAP signaling pathway (Wang et al., 2020; Ren et al., 2023).

According to the ceRNA hypothesis, hsa_circ_0075436 and hsa_circ_0005729 may function as molecular sponges for these miRNAs, thereby alleviating miRNA-mediated suppression of target genes such as CAMK2A, TREML4, COL3A1, and COL1A1. Using qRT-PCR, we confirmed that mRNA levels of COL1A1 in PBMCs from AIS patients were significantly lower than those in healthy controls. COL1A1 are key components of the extracellular matrix (ECM) and play essential roles in maintaining BBB integrity and facilitating tissue remodeling (Feng et al., 2021). Encapsulation of COL1A1 mRNA has been shown to promote ECM formation in photoaged skin (You et al., 2023). The reduced expression of COL1A1 in PBMCs may be related to BBB disruption observed in AIS patients in our study. In addition, the elevated COL1A1 expression has been reported to be associated with fibroblast and activation of CD8^+^ T cells and macrophages in the vascular wall of aortic aneurysm (Wagenhauser et al., 2024; Xie et al., 2024). Therefore, the downregulation of COL1A1 observed in our study may reflect an altered immune state in PBMCs, possibly linked to peripheral immune suppression. Following stroke, peripheral immune cells are rapidly activated and recruited to the injury site, followed by a phase of peripheral immune suppression that increases susceptibility to systemic infections (Zera and Buckwalter, 2020). Taken together, our findings suggest that hsa_circ_0075436 and hsa_circ_0005729 may contribute to BBB degradation and peripheral immune suppression after stroke through regulation of COL1A1 expression. Further mechanistic studies are needed to validate these interactions and evaluate their therapeutic potential.

To assess the diagnostic utility of these circRNAs, we performed ROC analysis, which confirmed that both circRNAs could distinguish AIS patients from healthy controls. Moreover, multivariate logistic regression analysis showed that reduced expression of hsa_circ_0075436 and hsa_circ_0005729, along with elevated blood glucose, hypertension, and atrial fibrillation, were independently associated with increased AIS risk. In contrast, higher lymphocyte counts and elevated circRNA levels were associated with a lower risk. These findings reinforce the multifactorial nature of AIS and highlight the importance of managing common risk factors such as hypertension, atrial fibrillation and glucose levels, which play a central role in stroke recurrence and prognosis (Maida et al., 2022).

Despite these promising findings, our study has several limitations. First, inconsistencies in circRNA annotation and naming conventions may lead to the omission of relevant candidates during data integration, potentially limiting comprehensiveness. Second, the study was conducted at a single center with a relatively homogeneous population, which may introduce selection bias and limit generalizability. Multicenter studies with larger, more diverse populations are necessary to validate and extend these findings. Third, the biological functions of hsa_circ_0075436 and hsa_circ_0005729 remain to be fully elucidated. Functional validation through *in vitro* and *in vivo* studies is critical to identify their precise roles in AIS pathology.

## 5 Conclusion

In summary, we profiled circRNA expression in PBMCs of AIS patients and constructed a ceRNA regulatory network to explore their functional implications. Two circRNAs—hsa_circ_0075436 and hsa_circ_0005729—were significantly downregulated in AIS, correlated with stroke severity, and appear to modulate key genes implicated in BBB disruption and peripheral immune suppression. These circRNAs hold promise as novel diagnostic and prognostic biomarkers. Our findings offer new insights into the molecular mechanisms underlying AIS and pave the way for future studies aimed at improving early diagnosis and precision therapy.

## Data Availability

The raw sequencing data presented in the study are deposited in the NCBI Sequencing Read Archive (SRA), accession number PRJNA1330736.

## Glossary

AIS: Acute ischemic stroke
MRI: Magnetic resonance imaging
CT: Computed tomography
CircRNAs: Circular RNAs
PBMCs: Peripheral blood mononuclear cells
W.HTSeq: Whole-transcriptome sequencing
DECs: Differentially expressed circRNAs
qRT-PCR: Quantitative real-time PCR
GO: Gene ontology
KEGG: Kyoto encyclopedia of genes and genomes
PPI: Protein–protein interaction
ceRNA: Competing endogenous RNA
NIHSS: National institutes of health stroke scale
PBS: Phosphate-buffered saline
rRNA: Ribosomal RNA
mRNA: Messenger RNA
miRNA: MicroRNA
UTRs: Untranslated regions
cDNA: Complementary DNA
ROC: Receiver operating characteristic
FPR: False positive rate
TPR: True positive rate
FP: False positives
TN: True negatives
AUC: Area under the curve
FC: Fold change
OR: Odds ratios
CI: Confidence Interval
DAMPs: Damage-associated molecular patterns
BBB: Blood-brain barrier
ECM: Extracellular matrix
IQR: Interquartile range
NLR: Neutrophil-to-lymphocyte ratio
PLR: Platelet-lymphocyte ratio
FDR: False discovery rate
GLU: Glucose
