# Supplementary material for: Peripheral circular RNAs hsa_circ_0075436 and hsa_circ_0005729 as diagnostic and prognostic biomarkers in acute ischemic stroke: expression profiles and mechanistic insights
Source: Front Mol Biosci. 2025 Oct 1;12:1657284. doi: 10.3389/fmolb.2025.1657284 (PMC12520956; doi:10.3389/fmolb.2025.1657284)
Supplement: Supplementary file 1 [file DataSheet1.zip › Supplementary files/Supplementary_Material.docx]

Supplementary Material

# Supplementary Figures and Tables

## Supplementary Figures


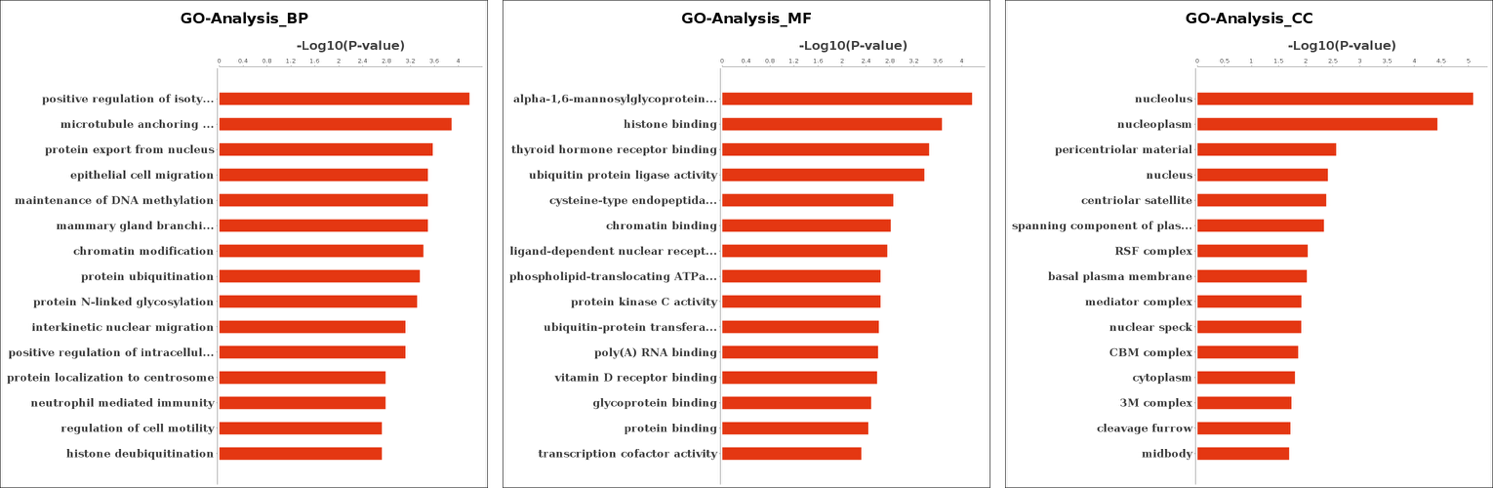
**Supplementary Figure S1.** GO enrichment analysis diagram of host genes for the top 10 DECs.


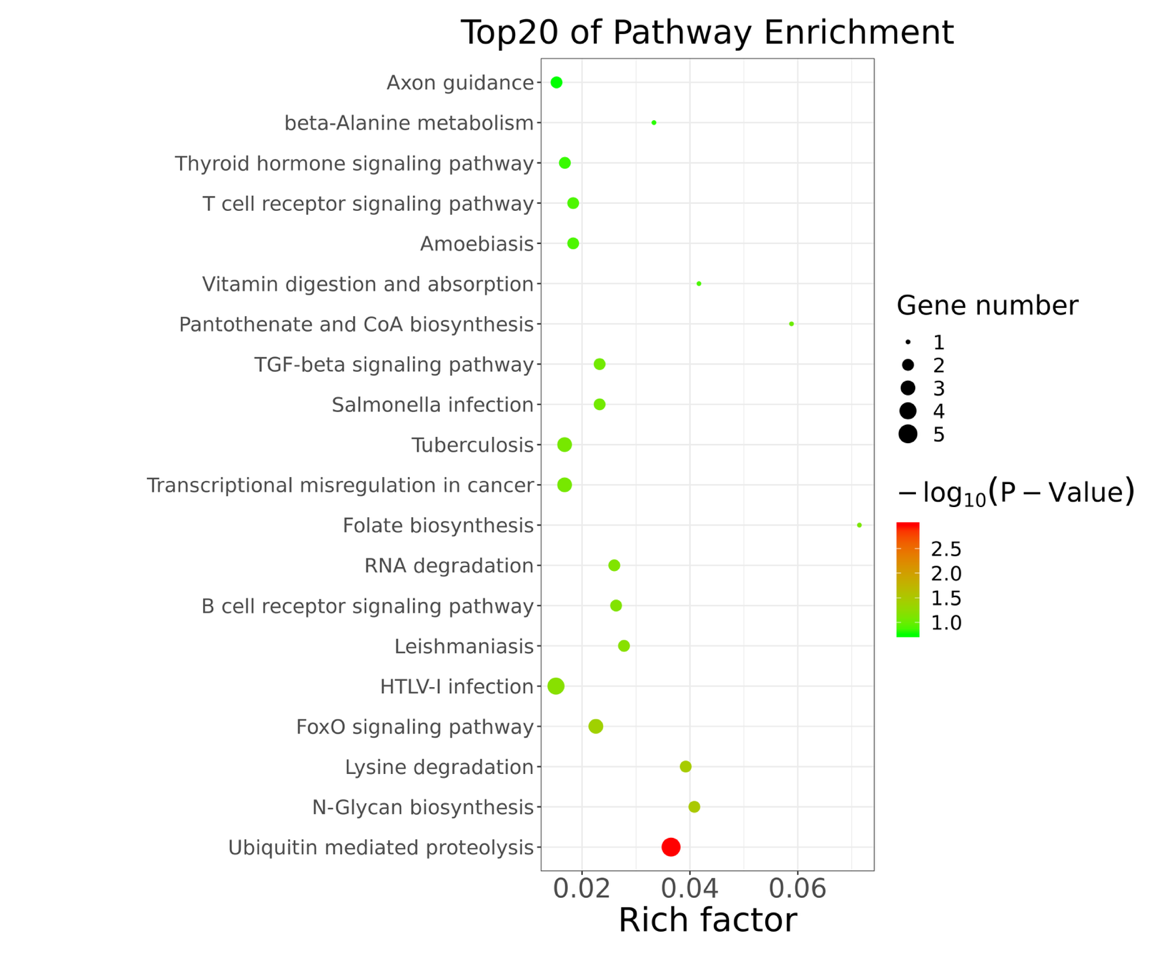


**Supplementary Figure S2.** KEGG enrichment analysis diagram of host genes for the top 10 DECs.


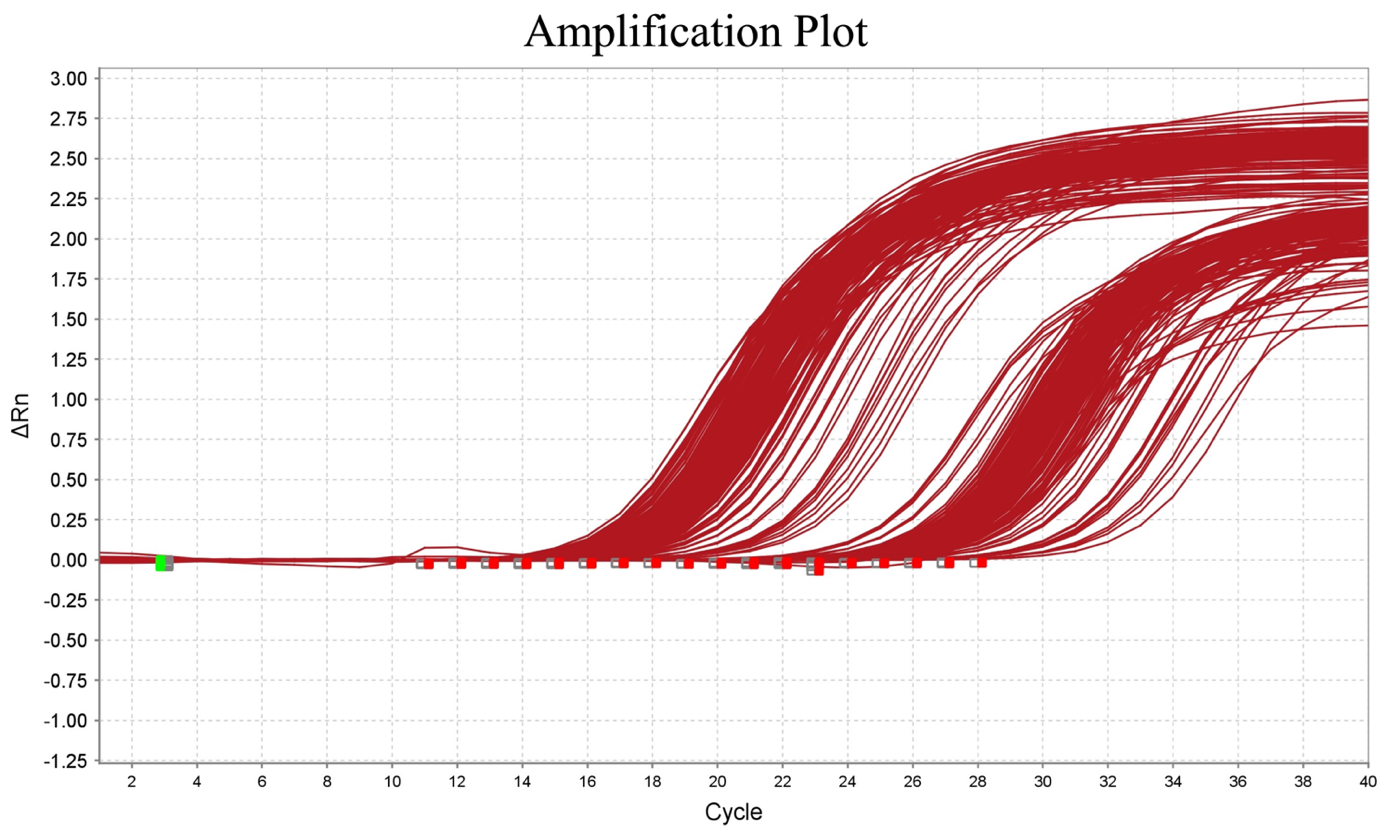


**Supplementary Figure S3.** Amplification Plot of qRT-PCR for has_circ_0005729 and has_circ_0075436 (partial samples).


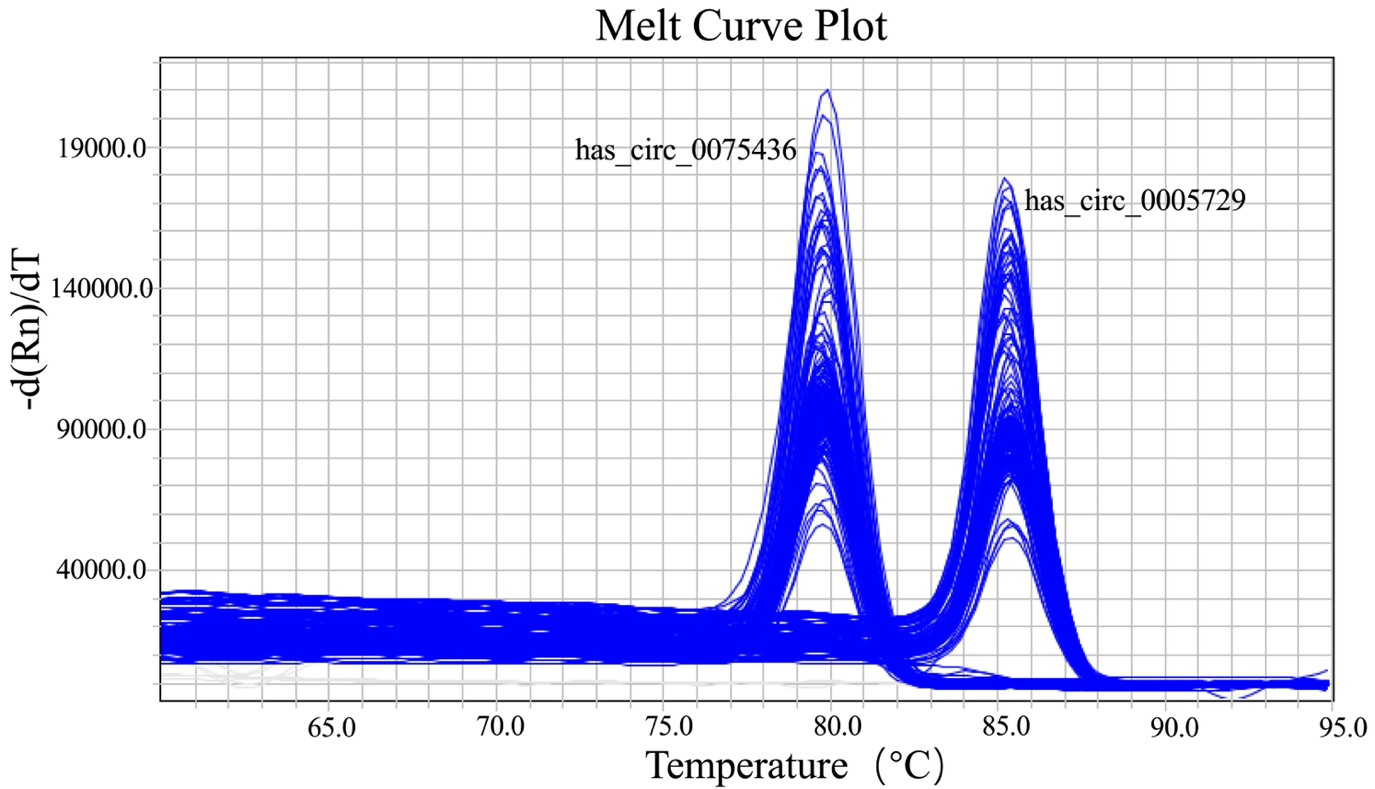


**Supplementary Figure S4.** Melt curve Plot of qRT-PCR for has_circ_0·005729 and has_circ_0075436 (partial samples)

## Supplementary tables

**Supplementary Table S1.** hsa-miR-1301-3p mimics and mimics NC used in this study.

| **miRNA mimics** | **Sequence (5'→3')** |
| --- | --- |
| hsa-miR-1301-3p mimics | UUGCAGCUGCCUGGGAGUGACUUC |
| mimics NC | UUGUACUACACAAAAGUACUG |

^1^ NC: Negative control.

**Supplementary Table S2.** Demographic characteristics of AIS group and control group.

| **Characteristics** | **AIS (*n* = 5)** | **Control (*n* = 5)** |
| --- | --- | --- |
| Age (mean ± SD), year | 54.8 ± 13.41 | 56.2 ± 13.29 |
| Male, n (%) | 3 (60%) | 3 (60%) |
| Hypertension, n (%) | 4 (80%) | 3 (60%) |
| Diabetes, n (%) | 2 (40%) | 2 (40%) |
| Coronary heart disease, n (%) | 0 (0%) | 0 (0%) |
| GLU (mmol/L) | 7.54 ± 2.74 | 6.18 ± 1.96 |
| TC (mmol/L) | 5.32 ± 2.38 | 5.34 ± 0.54 |
| LDL (mmol/L) | 3.52 ± 1.93 | 3.36 ± 0.34 |

^2^ Values are expressed as mean ± standard deviation or n (%). GLU: Glucose; TC:

Total cholesterol; TG: Triglycerides; LDL: Low density lipoprotein.

**Supplementary Table S3.** The primer sequences of 10 differentially DECs.

| **CircRNA primer** | **Sequence (5'→3')** | **length (bp)** |
| --- | --- | --- |
| *hsa_circ_0005376*-F | CCTGATGTGAACCTTATTGGGGA | 257 |
| *hsa_circ_0005376*-R | TTTCATCAAAATATGCAGGATCTCTT |  |
| *hsa_circ_0003039*-F | GCCTCCTCAAGTTATTCAGTCTCG | 272 |
| *hsa_circ_0003039*-R | GTTCAGCAAAGAGAGCAGAATAGGAA |  |
| *hsa_circ_0069249*-F | GATGGGACTCCTGTGGGTT | 331 |
| *hsa_circ_0069249*-R | TCAGCCTGCTCAGTGAACGA |  |
| *hsa_circ_0000239*-F | TCAAATTGCTGCCATATTAGACCAA | 252 |
| *hsa_circ_0000239*-R | GATCACATTCAGGCCAACCAG |  |
| *hsa_circ_0036372*-F | TGACCCTGATAGTCCTTTGCACA | 704 |
| *hsa_circ_0036372*-R | GGTGAAGAAGATGGATAGGATTCCC |  |
| *hsa_circ_0005729*-F | ATATGGTGTTTCTTCTATCATTCCAA | 282 |
| *hsa_circ_0005729*-R | TTTTCAAGGTCTAAGGCCCGTTC |  |
| *hsa_circ_0114376*-F | CAGCAGCACTAACAGGGGGAT | 937 |
| *hsa_circ_0114376*-R | TCTGCTGCACCATTGTATCTGAA |  |
| *hsa_circ_0044949*-F | GAATGCACATGACACCACAAAGACA | 1259 |
| *hsa_circ_0044949*-R | AGCATTCTTTCCATTTCTTCCCGTA |  |
| *hsa_circ_0008460*-F | CACAGCTATACCAAACTTAAAGTGTTC | 789 |
| *hsa_circ_0008460*-R | TGCCTGCTTCATCTTTATGCAC |  |
| *hsa_circ_0075436*-F | CTACGTGAAAGATCTGAAAGGAGCA | 450 |
| *hsa_circ_0075436*-R | AGAAACTTAAATCGCTGAAGCAC |  |

^3^ Bp: Base pair; CircRNA: Circular RNA; F: Forward; R: Reverse.

**Supplementary Table S4.** Whole-Transcriptome sequencing results of has_circ_0005729 and has_circ_0075436 target genes

| **mRNA** | **Log2(FC)** | **FDR** | **Regulate** |
| --- | --- | --- | --- |
| CAMK2A | -11.79084554 | 0.0000 | down |
| CHN1 | -1.294844852 | 0.02128 | down |
| COL1A1 | -4.924380116 | 0.00026 | down |
| COL3A3 | -4.666554066 | 0.00130 | down |
| TREML4 | -4.532469874 | 0.03415 | down |

^4^ FC: fold change; FDR: False discovery rate.

**Supplementary Table S5.** The primer sequences of *hsa_circ_0075436* and *hsa_circ_0005729* target genes.

| **mRNA primer** | **Sequence (5'→3')** | **length (bp)** |
| --- | --- | --- |
| *CAMK2A*-F | AAGAAGTTCAATGCCAGGAGGAA | 143 |
| *CAMK2A*-R | ATGGTGGTGTTGGTGCTCTC |  |
| *TREML4*-F | CCCAACCACGTCTCCTATGTG | 173 |
| *TREML4*-R | ATAGCACCAAGACCAGGAATCT |  |
| *COL1A1*-F | TCGAGGGCCAAGACGAAGA | 114 |
| *COL1A1*-R | CGTTGTCGCAGACGCAGAT |  |
| *COL3A1-*F | GGCTACTTCTCGCTCTGCTT | 125 |
| *COL3A1*-R | TTGGCATGGTTCTGGCTTCC |  |

^4^ Bp: Base pair; F: Forward; R: Reverse.
