# Supplementary figures and images for: Peripheral circular RNAs hsa_circ_0075436 and hsa_circ_0005729 as diagnostic and prognostic biomarkers in acute ischemic stroke: expression profiles and mechanistic insights
Source: Front Mol Biosci. 2025 Oct 1;12:1657284. doi: 10.3389/fmolb.2025.1657284 (PMC12520956; doi:10.3389/fmolb.2025.1657284)

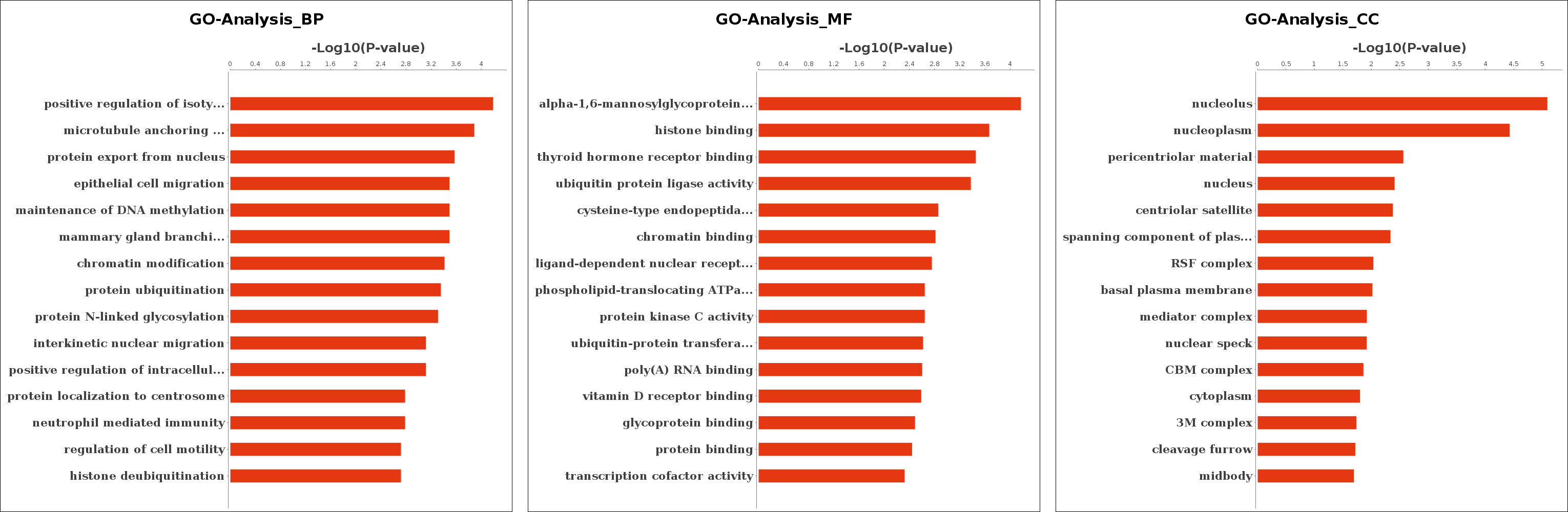

Supplement: Supplementary file 1 [file DataSheet1.zip › Supplementary files/Supplementary Figure S1.tiff]

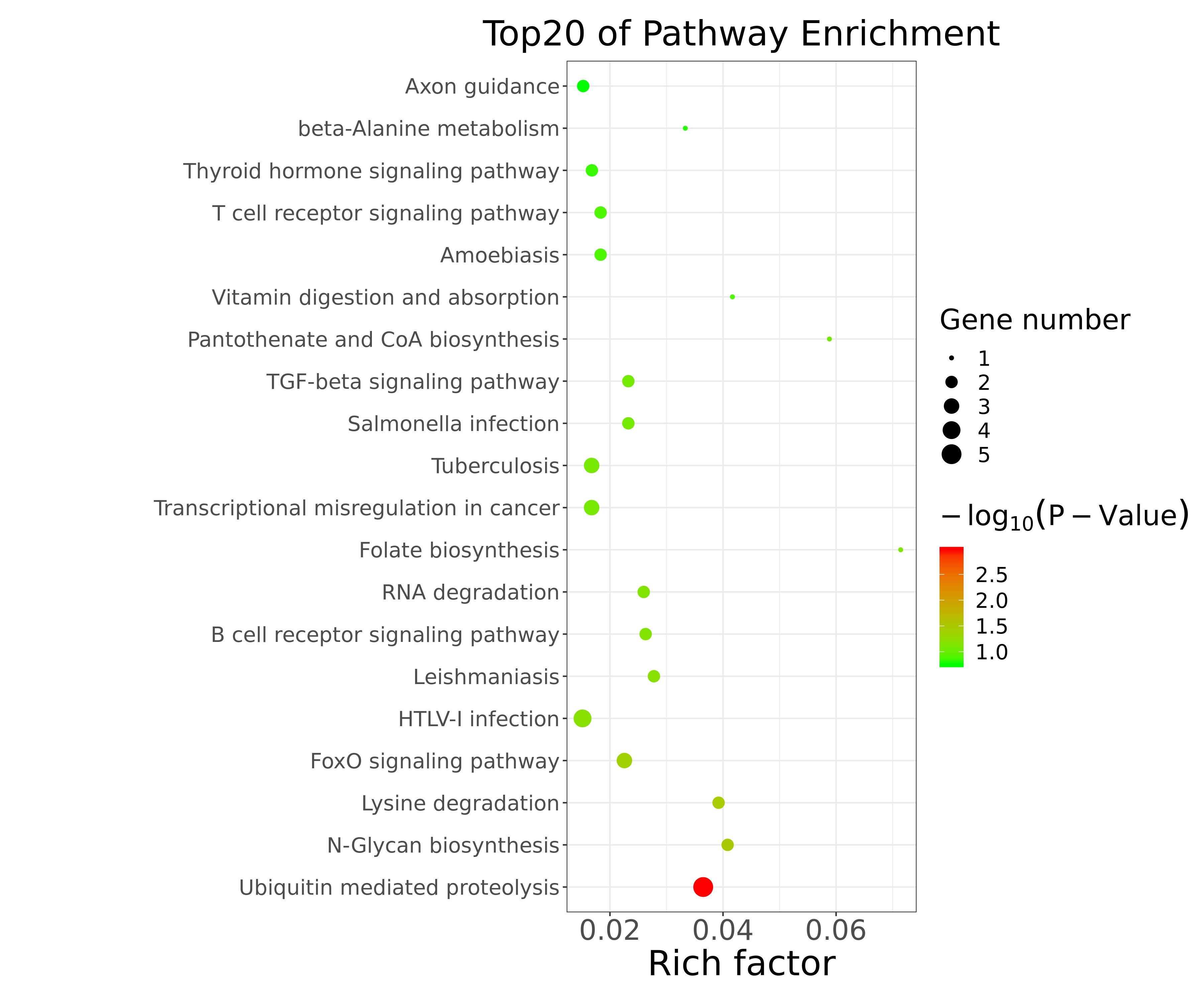

Supplement: Supplementary file 1 [file DataSheet1.zip › Supplementary files/Supplementary Figure S2.tiff]
